# Ultrasonic Scalpel vs. Polymeric Clip Laparoscopic Varicocelectomy in Adolescents with Symptomatic Varicocele

**DOI:** 10.3390/jcm13154322

**Published:** 2024-07-24

**Authors:** Zenon Pogorelić, Karlo Poljak, Miro Jukić, Katarina Vukojević

**Affiliations:** 1 Department of Surgery, School of Medicine, University of Split, 21000 Split, Croatia; 2 Department of Pediatric Surgery, University Hospital of Split, 21000 Split, Croatia; 3 Department of Anatomy, Histology and Embryology, School of Medicine, University of Split, 21000 Split, Croatia

**Keywords:** varicocele, laparoscopic varicocelectomy, varicocelectomy, ultrasonic scalpel, polymer ligating clip, adolescents, minimally invasive surgery, laparoscopic surgery

## Abstract

**Background:** The aim of this study was to investigate treatment outcomes in adolescents who underwent laparoscopic surgery with an ultrasonic scalpel for symptomatic varicocele compared with adolescents who underwent surgery with a polymer clip. **Methods:** A total of 270 adolescents with a median age of 16 (interquartile range, IQR 13–17) years were included in the study. Taking into account the laparoscopic varicocelectomy technique used, the patients were divided into two groups. In the first group (*n* = 151), a polymer clip was used, while in the second group (*n* = 119), an ultrasonic scalpel was used to resect the spermatic vessels. The primary outcome measure was the effect of the laparoscopic technique used on treatment outcomes (postoperative complications and recurrence rates). Secondary outcomes were the duration of surgery and anesthesia and the length of hospital stay. **Results:** The duration of the surgical procedure (12 min (IQR 11, 15) versus 15 min (12, 19), *p* = 0.029) and anesthesia (21.5 min (16, 29.5) versus 28 min (23, 34), *p* = 0.003) was shorter in the group of adolescents in whom laparoscopic varicocelectomy was performed with an ultrasonic scalpel than in the group in which a polymer clip was used. No statistically significant difference was found between the groups studied in terms of length of hospital stay, recurrence rate (*p* >0.999), and complications (*p* = 0.703). There were no cases of testicular atrophy in either group. In the group of patients who underwent laparoscopic varicocelectomy with an ultrasonic scalpel, a slightly higher incidence of hydroceles was found (*n* = 4, 3.4%) than in the group in which a polymer clip was used (*n* = 2, 1.3%) (*p* = 0.410). At six-month follow-up, it was found that the majority of patients showed moderate or significant improvement in the spermogram after laparoscopic varicocelectomy (*n* = 85, 89.5%). In addition, the subjective discomfort or pain disappeared in the majority of patients (*n* = 71, 93.4%). The testicular volume increased significantly in 132 adolescents (89.8%). **Conclusions:** Laparoscopic varicocelectomy with a polymer clip or ultrasonic scalpel is safe and effective in adolescents with symptomatic varicocele. Treatment outcomes after laparoscopic varicocelectomy are the same regardless of whether a polymer clip or an ultrasonic scalpel is used to resect the spermatic vessels. The use of an ultrasonic scalpel for resection of the spermatic vessels shortens the overall duration of surgery and anesthesia.

## 1. Introduction

The term varicocele represents dilated, enlarged, and tortuous veins in the pampiniform plexus of the scrotum and is considered the most common cause of male infertility [1,2,3]. It is seen less frequently under the age of 10. After this age, the incidence increases [3,4] so that the overall incidence of varicocele in children and adolescents is 10–15%, and 40% of men are treated for infertility [1,2,3,4,5,6,7,8]. Elimination of varicocele improves semen parameters in over 50% of men [5,8]. Left-sided varicocele is the most common (90%), and 10% of varicoceles are bilateral. A sole right-sided varicocele is an infrequent condition [3].

The treatment of varicocele in the pediatric population is still controversial, as are the correct indications for it [5]. Indications that are still considered appropriate in the pediatric population today are abnormal spermogram findings (adolescents > 16 years of age), progressively and consistently elevated FSH/LH levels, progressive testicular volume discrepancy (>20% decrease in left testicular volume) that worsens over time, and subjective sensation of pain and persistent discomfort on the affected side of the testis [5].

Numerous studies have described several surgical techniques, including various types of open varicocelectomy and minimally invasive ones, such as microsurgical, microscopic laparoscopic, laparoscopic, or robotic-assisted varicocelectomy [1,2,3,4,5,6,7,8,9,10]. Minimally invasive surgery and laparoscopic varicocelectomy have gained popularity and acceptance among pediatric surgeons and urologists [5]. In addition, the benefits of laparoscopic procedures are well-known and have been reported multiple times in the literature, such as lower pain and length of hospital stay, faster recovery, fewer complications, and better cosmesis [5]. Despite all this, there is still no consensus on which varicocele surgical technique is the gold standard.

The safe use of polymeric clips to secure the spermatic vessels has been repeatedly reported [3,5,7,11,12]. The effective use of ultrasonic scalpels in open and endoscopic surgery of vascular structures has already been described [10,13,14]. Polymer ligating clips can be used in all laparoscopic procedures to ligate blood vessels (e.g., in laparoscopic splenectomy, gastrectomy, nephrectomy, colectomy, etc.), cystic duct in laparoscopic cholecystectomy, or even to supply the base of the appendix in laparoscopic appendectomy [15,16,17,18].

The use of resterilized or/and reprocessed single-use devices in medicine and laparoscopic surgery has been described and plays a major role in reducing medical waste and improving the environmental impact of human nature and costs for the healthcare industry [13,19,20].

This study focuses on the comparison between laparoscopic varicocelectomy, which differs only in securing and transecting the spermatic vessels suprainguinally, approximately 1 cm above the internal inguinal ring, using either a polymer clip and scissors or a reused ultrasonic scalpel.

This study aimed to determine and compare the treatment outcomes, postoperative results, long-term follow-up, and complication rates between the two groups studied and to evaluate the use and safety of ultrasonic scalpels in laparoscopic varicocelectomy. In addition, the demographic and clinical characteristics and indications for surgery were investigated.

## 2. Methods

### 2.1. Patients

**Figure 1:**
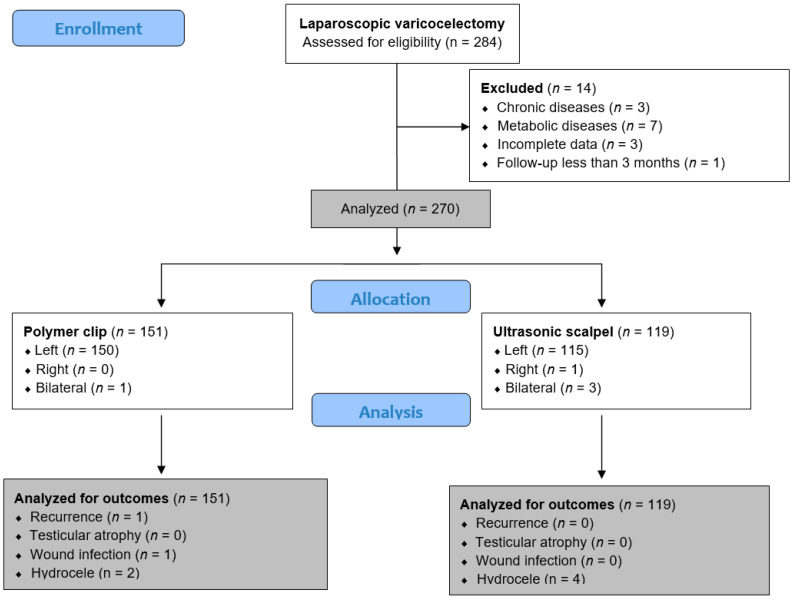
Flowchart of the study.

All adolescents who underwent laparoscopic varicocelectomy at the Department of Pediatric Surgery at the University Hospital of Split between 1 May 2019 and 1 May 2024 were included in the analysis. This study was designed as a retrospective trial with a prospective arm. The data of the patients who underwent varicocelectomy with a polymer clip were collected retrospectively, while the data of the patients who underwent varicocelectomy with an ultrasonic scalpel were collected prospectively. During the study period, a total of 284 adolescents who underwent laparoscopic varicocelectomy were identified. However, 14 adolescents were excluded from further analysis because they met one or more exclusion criteria. Finally, 270 adolescents met the inclusion criteria and were analyzed further. Male adolescents aged 13 to 17 years who underwent laparoscopic surgery at our institution for the treatment of symptomatic varicocele using an ultrasonic scalpel or polymer clip and who were followed up for at least three months after surgery were included in the study. Adolescents who underwent open retroperitoneal or inguinal/subinguinal microsurgical varicocelectomy, adolescents with chronic, metabolic, and endocrinologic diseases or systemic infections, and those with incomplete data in the medical records were excluded from the study. A flow chart of the study is shown in Figure 1.

### 2.2. Ethical Aspects

This study followed the Declaration of Helsinki of the World Medical Association and its subsequent amendments or comparable ethical standards. The Institutional Review Board of the University Hospital of Split approved the study (approval number: 500-03/23-01/227; date of approval: 27 November 2023).

### 2.3. Outcomes of the Study

The primary outcome measure was the occurrence of complications (hydrocele, recurrence, or testicular atrophy) and the analysis of treatment outcomes between the two surgical techniques of laparoscopic varicocelectomy. Secondary outcome measures were the rate of reoperations, number of readmissions within 30 days (ReAd) [21], duration of the operation, and length of hospital stay.

### 2.4. Study Design

Hormones (FSH and LH), color Doppler ultrasound of the testes and seminal vessels, and semen analysis in adolescents aged ≥ 16 years were performed for each patient before the treatment decision. The indications for varicocelectomy corresponded to the current guidelines of the European Association of Urology (EAU): testicular hypotrophy (size difference >20%), symptomatic varicocele, bilateral varicocele, and pathological sperm quality on semen analysis [22].

The patients were divided into two study groups concerning the technique used to supply spermatic blood vessels during laparoscopic varicocelectomy. In the first group (*n* = 151), a non-absorbable polymer clip was used for laparoscopic varicocelectomy, while in the second group of patients (*n* = 119), a reprocessed ultrasonic scalpel was used for varicocelectomy. The decision as to which technique was used for spermatic blood vessel resection was based on the operating surgeon’s preference.

The following data were collected for each patient enrolled in the study: age, height, body weight, body mass index (BMI), lateralization and degree of varicocele, indications for surgery, early and late complications, recurrence rate, reoperation rate, ReAd, duration of surgery/anesthesia, and length of hospital stay.

### 2.5. Surgical Technique

**Figure 2:**
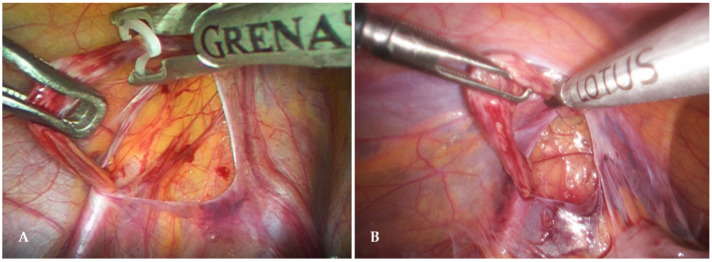
Laparoscopic varicocelectomy—(**A**) Application of polymer clip; (**B**) Ultrasonic scalpel varicocelectomy.

In all adolescents, general anesthesia was used for laparoscopic varicocelectomy. The patient was placed in the supine position. A 5 mm incision was made supraumbally. A Veress needle was used, and a pneumoperitoneum of 10–12 mmHg was achieved. Once the pneumoperitoneum was reached, an initial 5 mm trocar for the camera was inserted through the supraumbilical incision. After exploring the abdominal cavity, two additional 5 mm trocars were placed 1–2 cm below the umbilicus in the left and right clavicular lines along the lateral edge of the rectus abdominis muscle. After identifying the vas deferens and spermatic blood vessels, the peritoneum was opened using laparoscopic scissors approximately one centimeter above the internal inguinal ring. After the spermatic blood vessels were isolated and separated from the lymphatic vessels, they were supplied with two polymer clips (Ligating Clips ML; Grena, Brentford, UK) (Figure 2A) and cut with endoscopic scissors between the two clips, or a reprocessed ultrasonic scalpel was used to resect the spermatic blood vessels (Lotus, BOWA-electronic GmbH, Gomaringen, Germany) (Figure 2B). At the end of the procedure, the intra-abdominal pressure was lowered to 5–6 mmHg to inspect the surgical site and confirm that there was no bleeding. At the end of the procedure, trocars are removed and the skin is closed with simple sutures.

### 2.6. Postoperative Protocol and Follow-Up

The majority of cases were performed as day surgery, and patients were discharged within 24 h. Oral intake was started two hours after surgery. Ibuprofen (Brufen, Mylan, Zagreb, Croatia) at a dose of 10 mg/kg was administered for pain relief. The criteria for discharge were good patient condition, tolerance of oral intake, and pain control. After discharge, each patient was examined as an outpatient. At the first follow-up examination after 7 days, the stitches were removed. Subsequently, patients were followed up on an outpatient basis after 1, 6, and 12 months to evaluate the treatment results and detect late complications.

### 2.7. Statistical Analysis

The collected data were entered into the software packages Microsoft Office (version 16.74) for word processing and Microsoft Excel for the creation of tabular presentations. The Statistical Package for Social Sciences (SPSS, version 28.0, IBM Corp., Armonk, NY, USA) was used for statistical analysis. The Kolmogorov–Smirnov test was used to test the normality of the data distribution. Normally distributed continuous variables were expressed as mean and standard deviation (SD), while median and interquartile range (IQR) were used to express data that were not normally distributed. Categorical variables are described as absolute numbers and percentages. The *t*-test for independent samples or the Mann–Whitney U test was used to compare continuous variables, depending on the normal distribution of the data. The chi-square test was used to compare categorical variables. In cases where the frequency of each variable was low, Fisher’s exact test was used. The level of statistical significance was set at *p* < 0.05.

## 3. Results

During the 5-year study period, a total of 270 laparoscopic varicocelectomies were performed for symptomatic varicocele. Of these, polymer clips were used in 151 (55.9%) patients, while in the remaining 119 (44.1%) cases, a reprocessed ultrasonic scalpel was used to resect the spermatic blood vessels. The median age of all the patients was 16 years (IQR 15, 17). In the majority of cases in both groups, the varicocele was found on the left side (*n* = 265; 98.2%) with a median diameter of the veins of 3.5 mm (IQR 3.1, 4). The majority of cases were classified as grades II (*n* = 123; 45.5%) and III (*n* = 135; 50%). The most common indication for surgery in both groups was testicular hypotrophy (*n* = 147; 54.4%), followed by an abnormal spermogram (*n* = 90; 35.2%) and subjective discomfort or pain (*n* = 76; 28.1%). Only in four cases (1.5%) was a bilateral varicocele an indication for surgery. Both groups were symmetrical regarding the patients’ demographic and preoperative characteristics (Table 1).

No significant differences were found between the groups with regard to age (*p* = 0.423), height (*p* = 0.752), weight (*p* = 0.824), BMI (*p* = 0.741), frequency of comorbidities (*p* = 0.924), grade of varicocele (*p* = 0.071), or indication for surgery (*p* = 5.754). The only significant difference between the groups was found in relation to the preoperative vein diameter (*p* = 0.002), but this result was of no clinical significance.

The duration of the surgical procedure (12 min vs. 15 min, *p* = 0.029) and anesthesia (21.5 min vs. 28 min, *p* = 0.003) was statistically significantly shorter in the group of patients who underwent varicocelectomy with the ultrasonic scalpel than in the patients in whom a polymer clip was used. No statistically significant differences were found between the groups in terms of length of hospital stay (*p* > 0.999), recurrence rate (*p* > 0.999), and complications (*p* = 0.703). There were no cases of testicular atrophy in either group. A slightly higher incidence of postoperative hydrocele was observed in the patients in whom an ultrasonic scalpel (*n* = 4, 3.4%) was used compared to the patients in whom polymer clips (*n* = 2, 1.3%) were used (*p* = 0.410) (Table 2).

Six months after the procedure, the majority of patients who underwent laparoscopic varicocelectomy showed moderate or significant improvement in the spermogram (*n* = 85, 89.5%), with no improvement observed in only 10 (10.5%) patients. Subjective discomfort or pain disappeared in most patients (*n* = 71, 93.4%), five patients (6.6%) still reported pain or discomfort. An improvement in testicular volume was observed in 132 patients (89.8%). Clinical improvement was observed in all patients who underwent laparoscopic varicocelectomy for bilateral varicocele. No significant differences were found between the groups in terms of the postoperative treatment outcomes (Table 3).

## 4. Discussion

This study showed no significant differences in treatment outcomes and the incidence of postoperative complications between patients who underwent laparoscopic varicocelectomy with an ultrasonic scalpel or a polymer clip. The incidence of postoperative hydrocele was slightly higher in the group operated on with the ultrasonic scalpel, but not statistically significant. The postoperative recovery of the adolescents was uneventful, regardless of the varicocelectomy technique used. The incidence of reoperations and readmissions to the hospital was equal in both groups. In addition, the duration of surgery and anesthesia in the group of patients who underwent varicocelectomy with an ultrasonic scalpel was significantly shorter than that in the group treated with a polymer clip.

In a meta-analysis, Tandon et al. identified 1910 studies that covered most surgical techniques for treating varicocele. This meta-analysis aimed to evaluate the clinical outcomes of different surgical approaches in patients. The recurrence incidence was between 2.1% and 7.6% (recurrence is more common with embolization or sclerotherapy procedures). Testicular atrophy was not observed in any of the embolization, sclerotherapy, or laparoscopic techniques. Postoperative hydrocele varies between 0.8% and 11.4% and occurs frequently during laparoscopic varicocelectomy. The laparoscopic approach has been reported as a quick, safe, and relatively simple surgical technique for the treatment of varicocele [23].

Several studies have investigated high-energy devices for vessel sealing in laparoscopic varicocelectomy. From five different studies on laparoscopic varicocelectomy using high-energy devices for spermatic vessel sealing, mostly LigaSure, it was reported that the average duration ranged from 15 to 43.5 min [8,24,25,26,27,28,29]. The study by Koyle et al. also found that the duration of the procedure shortened after refinement of the technique, reducing the average duration to 14.6 min in the last two years of the study [24]. This is very similar to our results; in patients for whom the ultrasonic scalpel was the technique of choice, the average duration of the procedure was 12 min. In their study, Sasagawa I et al. also investigated the treatment results after laparoscopic varicocelectomy with an ultrasonic scalpel and found similar results to our study in terms of treatment outcomes and complications. The only difference is that the mean duration of the procedure in their study was 35 min, while the mean duration of laparoscopic varicocelectomy in our study was significantly shorter at 15 min [10].

The median length of hospital stay in this study was 24 h, the same regardless of the technique used, although the only complication reported in ultrasonic scalpel varicocelectomy was the formation of hydrocele with an incidence of 3.4%. In several other studies, the duration of hospitalization after varicocelectomy with high-energy devices for vessel sealing was reported to be between 21 and 31 h [25,26,29,30]. In contrast, another study that examined 72 patients reported a hospital stay of three to five days was reported [27]. The rate of postoperative hydrocele has been reported to range from 5% to 18% in several studies investigating high-energy devices for vessel sealing [24,25,26,27,29,30]. In addition, recurrent varicocele has been reported postoperatively, with an incidence of 0–3.8% [8,24,25,29].

Another technique observed in the current study, laparoscopic varicocelectomy with a polymer clip, is a safe, simple, and cost-effective method for the treatment of varicocele [5]. Several studies have investigated the polymeric ligating clips used in laparoscopic varicocelectomy. The average operation time was reported in several studies to be approximately 29 min [31,32,33]. In contrast, a study by Pogorelić et al. reported an average time of 12 min [5]. In comparison, the duration of the surgical procedure in this study for laparoscopic varicocelectomy using a polymer clip was 15 min. As mentioned above, the same median hospitalization time of 24 h was reported in a group of patients treated with a ligating polymer clip as in the other group treated with an ultrasonic scalpel. In addition, three cases of postoperative complications occurred in this group: one case of wound infection with an incidence of 0.7% and two cases of hydrocele development with an incidence of 1.3%. In the group treated with a ligating polymer clip, there was also one case of recurrence, with an incidence of 0.7%. Other studies that also reported patients treated with the ligating clip technique showed a similar length of hospital stay, with a mean of 24 h [5,31,32,33]. The incidence of complications varied between the studies analyzed; for example, the incidence of postoperative hydrocele was reported to be between 0–8.3% [5,30,31,32,33]. In contrast, the incidence of recurrence was usually 0%, although in a study published by Kachrilas et al., the incidence was about 10.4% [34]. As for wound hematomas or infections as complications of laparoscopic varicocelectomy, the incidence varies between 0.8% and 1.7% [5,32]. Ramírez Calazans A, et al. in their recently published study compared varicocelectomy using vessel sealing devices and polymer clips and found similar results to our study. They concluded that laparoscopic varicocelectomy is safe and effective for symptomatic varicocele in pediatric patients, regardless of the vascular occlusion technique used [33].

Since the treatment results are similar in both groups studied, the question arises as to whether it is justified to use the ultrasonic scalpel, considering that its price exceeds the price of the polymer clips many times over. A recent study has clearly shown that the reprocessed ultrasonic scalpel is as effective and safe to use as the new one [13]. The reuse of the ultrasonic scalpel, which would otherwise be thrown away, can, therefore, be a good argument in favor of laparoscopic varicocelectomy because it shortens the duration of the operation and leaves no foreign material in the body.

Preoperative treatment is of great importance for the selection of patients for surgery and the avoidance of possible complications in adolescents who do not require surgical treatment. With a relatively high incidence in the male population of 14 to 20%, varicocele can lead to fertility problems in only about 20% of patients [3,5,34,35]. Of the indications, testicular hypotrophy was the most common in this study, followed by an abnormal spermogram, subjective discomfort or pain, and bilateral varicocele. A similar distribution of indications for varicocelectomy in adolescents has also been found in other studies [3,5,8,9,10,11,12].

This study has certain limitations. The most important limitation is the retrospective design of the study, which means that patient data that could be important for the results are not available. In addition, the collection of certain data relevant to the study, particularly the spermogram, could pose an ethical problem. Furthermore, the single-center study design means that the results may not be generalizable to the general population. Finally, there is no randomization, so selection bias in the study may be possible. Therefore, it is necessary to replicate the results of this study in larger, randomized, prospective, multi-center studies to obtain more reliable data that could be included in standardized protocols for laparoscopic treatment of varicocele at the global level.

## 5. Conclusions

Laparoscopic varicocelectomy with a polymer clip or ultrasonic scalpel is safe and effective in adolescents with symptomatic varicocele. The outcomes of treatment after laparoscopic varicocelectomy are the same regardless of whether a polymer clip or an ultrasonic scalpel is used for the resection of the spermatic vessels. The use of an ultrasonic scalpel for resection of the spermatic vessels shortens the overall duration of laparoscopic varicocelectomy and anesthesia.

## Figures and Tables

**Table 1:** Demographic and preoperative characteristics of the patients.

Variables	Group I	Group II	*p*
	Polymeric Clip (*n* = 151)	Ultrasonic Scalpel (*n* = 119)	
Age (years)	16	16	0.423 *
median (IQR)	(15, 17)	(15, 17)	
Height (cm)	181	183	0.752 *
median (IQR)	(161, 189)	(163, 191)	
Weight (kg)	69 ± 15.5	71 ± 12.3	0.824 ^†^
mean ± SD			
BMI (kg/m^2^)	21.3 ± 4.1	22.1 ± 3.2	0.741 ^†^
mean ± SD			
Comorbidities, *n* (%)	6 (4)	5 (4.2)	0.924 ^‡^
Diameter of veins (mm)	3.4 (3.1, 4)	3.7 (3.3, 4.2)	0.002 *
median (IQR)			
Varicocele grade, *n* (%)			0.071 ^§^
I	10 (6.6)	2 (1.7)	
II	72 (47.7)	51 (42.9)	
III	69 (45.7)	66 (55.4)	
Lateralization, *n* (%)			
Left	150 (99.3)	115 (96.6)	0.216 ^§^
Right	0 (0)	1 (0.8)	
Bilateral	1 (0.7)	3 (2.5)	
Indication for surgery, *n* (%)			
Abnormal spermiogram	49 (32.5)	46 (40)	0.307 ^‡^
Testicular hypotrophy	84 (55.6)	63 (53)	0.659 ^‡^
Subjective discomfort/pain	47 (31)	29 (24.4)	0.220 ^‡^
Bilateral varicocele	1 (0.7)	3 (2.5)	0.323 ^§^

* Mann–Whitney U test; ^†^ independent samples *t*-test; ^‡^ Chi-square test; ^§^ Fisher’s exact test; BMI—Body mass index; IQR—Interquartile range; SD—Standard deviation.

**Table 2:** Intraoperative data and treatment outcomes.

Variables	Group I	Group II	*p*
	Polymeric Clip (*n* = 151)	Ultrasonic Scalpel (*n* = 119)	
Duration of surgery (min) median (IQR)	15 (12, 19)	12 (11, 15)	0.029 *
Duration of anesthesia (min) median (IQR)	28 (23, 34)	21.5 (16, 29.5)	0.003 *
LOS (days); Median (IQR)	1 (1, 1)	1 (1, 1)	>0.999 ^†^
Recurrence, *n* (%)	1 (0.7)	0 (0)	>0.999 ^†^
Complications, *n* (%)			
Wound infection	1 (0.7)	0	>0.999 ^†^
Hydrocele	2 (1.3)	4 (3.4)	0.410 ^†^
Follow-up (months) Median (IQR)	29 (24, 33)	31 (26, 35)	0.651 *

* Mann–Whitney U test; ^†^ Fisher’s exact test; LOS—Length of hospital stay; IQR—Interquartile range.

**Table 3:** Postoperative treatment outcomes.

		Group I (Polymer Clip) (*n* = 151)		Group II (Ultrasonic Scalpel) (*n* = 119)		p *
Parameter	Outcome	*n*	%	*n*	%	
Spermogram		49		46		
	Moderate improvement	11	22.5	10	21.7	0.741 *
	Significant improvement	32	65.3	32	69.6	
	No improvement	6	12.2	4	8.7	
Subjective discomfort or pain		47		29		
	Moderate improvement	4	8.9	4	10.8	0.999 *
	Significant improvement	40	82.2	23	79.6	
	No improvement	3	8.9	2	9.6	
Testicular atrophy		84		63		
	Moderate improvement	10	13.8	8	11.6	0.813 ^†^
	Significant improvement	65	75.0	49	76.8	
	No improvement	9	11.2	6	11.6	
Bilateral varicocele		1		3		
	Moderate improvement	0	0	1	33.3	0.999 *
	Significant improvement	1	100	2	66.7	
	No improvement	0	0	0	0	

* Fisher’s exact test; ^†^ Chi-square test. Note: Some patients had more than one indication for surgical treatment.

## Data Availability

The data assessed and reported here can be obtained from the authors upon reasonable request following ethical and privacy principles.
